# Hematopoietic stem cell gene therapy targeting TGFβ enhances the efficacy of irradiation therapy in a preclinical glioblastoma model

**DOI:** 10.1136/jitc-2020-001143

**Published:** 2021-03-11

**Authors:** Tereza Andreou, Jennifer Williams, Rebecca J Brownlie, Robert J Salmond, Erica Watson, Gary Shaw, Alan Melcher, Heiko Wurdak, Susan C Short, Mihaela Lorger

**Affiliations:** 1 School of Medicine, University of Leeds, Leeds, UK; 2 Division of Radiotherapy and Imaging, Institute of Cancer Research, London, UK

**Keywords:** brain neoplasms, cell engineering, central nervous system neoplasms, immunotherapy, macrophages

## Abstract

Patients with glioblastoma (GBM) have a poor prognosis, and inefficient delivery of drugs to tumors represents a major therapeutic hurdle. Hematopoietic stem cell (HSC)-derived myeloid cells efficiently home to GBM and constitute up to 50% of intratumoral cells, making them highly appropriate therapeutic delivery vehicles. Because myeloid cells are ubiquitously present in the body, we recently established a lentiviral vector containing matrix metalloproteinase 14 (MMP14) promoter, which is active specifically in tumor-infiltrating myeloid cells as opposed to myeloid cells in other tissues, and resulted in a specific delivery of transgenes to brain metastases in HSC gene therapy. Here, we used this novel approach to target transforming growth factor beta (TGFβ) as a key tumor-promoting factor in GBM. Transplantation of HSCs transduced with lentiviral vector expressing green fluorescent protein (GFP) into lethally irradiated recipient mice was followed by intracranial implantation of GBM cells. Tumor-infiltrating HSC progeny was characterized by flow cytometry. In therapy studies, mice were transplanted with HSCs transduced with lentiviral vector expressing soluble TGFβ receptor II–Fc fusion protein under MMP14 promoter. This TGFβ-blocking therapy was compared with the targeted tumor irradiation, the combination of the two therapies, and control. Tumor growth and survival were quantified (statistical significance determined by t-test and log-rank test). T cell memory response was probed through a repeated tumor challenge. Myeloid cells were the most abundant HSC-derived population infiltrating GBM. TGFβ-blocking HSC gene therapy in combination with irradiation significantly reduced tumor burden as compared with monotherapies and the control, and significantly prolonged survival as compared with the control and TGFβ-blocking monotherapy. Long-term protection from GBM was achieved only with the combination treatment (25% of the mice) and was accompanied by a significant increase in CD8+ T cells at the tumor implantation site following tumor rechallenge. We demonstrated a preclinical proof-of-principle for tumor myeloid cell-specific HSC gene therapy in GBM. In the clinic, HSC gene therapy is being successfully used in non-cancerous brain disorders and the feasibility of HSC gene therapy in patients with glioma has been demonstrated in the context of bone marrow protection. This indicates an opportunity for clinical translation of our therapeutic approach.

## Background

There are around 11 700 new primary brain tumor cases in the UK every year and the incidence of cases is on the rise. The prognosis is worst for patients with glioblastoma (GBM), the most aggressive type of brain tumor, with an average survival time between 12 and 18 months.^1^ The current standard of care for patients with GBM includes debulking surgery, followed by chemotherapy (temozolomide) and irradiation.^2 3^

Elevated transforming growth factor beta (TGFβ) levels in glioma have been associated with high tumor grade and poor patient outcomes.^4^ TGFβ signaling has been implicated in invasion, tumor angiogenesis, maintenance of GBM stem-like cells and immunosuppression.^5 6^ Various approaches targeting TGFβ signaling have been therefore investigated preclinically, and several therapeutic agents are being tested in clinical trials in high-grade brain tumors, including the monoclonal antibody fresolimumab, the anti-sense oligonucleotide trabedersen, and the small molecule inhibitor of TGFβ receptor kinase galunisertib.^5 6^ Current preclinical and clinical data suggest that TGFβ blockade is likely to be most potent in combination with other treatments, including irradiation.^7–10^

Abnormal and poorly perfused tumor vasculature hinders an efficient delivery of therapies to the main glioma mass, while the blood–brain barrier is likely to hinder delivery of therapeutics to cancer cells typically invading the surrounding tissue.^1 3 11^ Thus, new approaches are urgently needed for the delivery of therapies to brain tumors. In this context, different types of stem cells have been shown to display tumor-homing properties. While neuronal and mesenchymal stem cells have been extensively studied in preclinical gene therapy targeting brain tumors,^12^ only a few studies have focused on hematopoietic stem cells (HSCs).^13 14^ In the clinic, HSC gene therapy has been successfully used in non-cancerous brain disorders, including adrenoleukodystrophy.^15 16^

We have recently demonstrated that the matrix metalloproteinase (MMP) 14 promoter drives gene expression specifically within the myeloid progeny of HSCs infiltrating brain metastases in contrast to the myeloid cells in other organs.^17^ Here, we used this promoter in HSC gene therapy to deliver TGFβ-blocking peptide to experimental GBM in combination with radiation therapy.

## Materials and methods

### Cell lines and primary cells

Firefly luciferase (Fluc)-tagged GL261 cells were obtained from Covance and HEK293 cells from ATCC. CT-2A cells were kindly provided by Dr David Stojdl and Charles Lefebvre (CHEO Research Institute, Ottawa) and stably transduced with pFUW-Fluc lentiviral vector.^18^ Cells were cultured in Dulbecco's modified eagle's medium (DMEM) containing 10% Fetal bovine serum (FBS), glutamine and penicillin/streptomycin. OT-I T cells were isolated from *Rag1-/-* OT-I CD45.1 mice^19^ and cultured in Iscove modified dulbecco medium (IMDM; Gibco), 10% FBS, Pen/Strep, 2 mM glutamine, 50 μM 2-Mercaptoethanol.

### Mice

Six to 8-week-old female C57Bl/6J mice were purchased from Charles River Laboratories, UK. C57Bl/6-Tg(UBCGFP)30Scha/J mice were purchased from Jackson Laboratories and bred at the University of Leeds. Animals were kept in individually ventilated cages in a specific-pathogen-free facility.

### Lentiviral expression constructs

The soluble transforming growth factor beta receptor II (sTGFβRII) fragment^20^ fused to a linker region (atatcggccatggtt) and the mouse Fc chain was gene synthesized (Genscript), subcloned into pF-MMP14-GW or pFUGW vectors^17^ using AgeI and EcoRI restriction sites, resulting in the pF-MMP14-sTGFβRIIFc and pFUW-sTGFβRIIFc lentiviral vectors, respectively. Lentiviral stocks were generated and lentiviral titres determined as previously described.^17^

### Transduction and transplantation of HSCs

HSCs were isolated from C57Bl/6J mice, transduced with lentiviral vectors, and transplanted into recipient mice as previously described.^17^

### Intracranial glioma models

GL261/Fluc or CT-2A/Fluc cells (1×10^5^ in 2 μL basal MEM medium) were implanted into mice brains and tumor growth was monitored by bioluminescence imaging as previously described.^17 18^ Survival studies were performed as previously described.^17^ One mouse from the control group (MMP14:GFP) was excluded from survival analysis due to the lack of tumor growth.

### Targeted irradiation

Prior to irradiation, mice were randomized based on the bioluminescence signal intensity. A fractionated dose of 5 Gy/day on three consecutive days was delivered using a Small Animal Research Radiation Platform (SARRP; Xstrahl). After cone beam CT scan, the correct segmentation was adjusted and the isocenter was aligned to the site of injection. The prescription dose selected and a single beam with a 3 mm×3 mm collimator was used. Muriplan was used to calculate the dose to medium value and obtain the irradiation time.

### Tissue dissociation and flow cytometry

Brain tumor tissue was enzymatically dissociated as previously described.^17^ The following antibodies were used: anti-CD45-PECy7 (30-F11; Biolegend), anti-F4/80-AF700 (CI:A3-1; BioRad), anti-CD11b-V450 (M1/70; BD Bioscience), anti-Gr1-PerCP (RB6-8C5; Biolegend), anti-Ly6C-Viogreen (1G7.G10; Miltenyi), anti-Ly6G-APC (1A8; Biolegend), anti-CD3e-APC-Vio770 (REA606; Miltenyi), anti-CD8b-PeCy7 (YTS156.7.7; Biolegend), and anti-CD4-APC (GK1.5; Miltenyi). The corresponding isotype control antibodies were from Biolegend, eBioscience or BD Bioscience. Samples were analyzed on LSRII (BD Biosciences), Cytoflex S or Cytoflex LX (Beckman Coulter) flow cytometers. Flow cytometry data were quantified with FACSDiva or CytExpert V.2.3 software.

### Cell transfection and western blot analysis

HEK293 cells were transiently transfected with pFUW-sTGFβRIIFc plasmid using Lipofectamine 2000 (Invitrogen). Cell lysates and cell culture supernatants (collected into serum-free medium for 48 hours) were prepared and analyzed by western blot as previously described,^17^ using anti-mouse TGFβ RII primary antibody (R&D Systems, AF532) and secondary anti-goat HRP antibody (Invitrogen). Anti-actin antibody (Sigma A1978) was used as a loading control. Equal cell numbers (for cell lysates) and equal volumes (for cell culture supernatants) were loaded for all samples.

### Inhibition of SMAD phosphorylation by sTGFβRIIFc

OT-I T cells (2×10^6^) isolated from lymph nodes were incubated with 5 ng/mL TGFβ in 1 mL complete medium for 30 min at 37°C, alone or in the presence of TGFβ inhibitor SB431542 (Sigma; 5 μM final concentration), cell culture supernatant collected from pFUGW or pFUW-sTGFβRIIFc-transfected HEK293 cells. Following fixation with 2% paraformaldehyde (PFA) and permeabilization with BD phosphoflow Perm buffer III, the mean fluorescence intensity of phospho-SMAD was quantified by flow cytometry, using anti-pSMAD2 (S465/467) SMAD3 (S423/425) rabbit mAb (Cell Signaling; D27F4), followed by anti-rabbit-PacificBlue secondary antibody.

HeLa cells were seeded in 6-well plates at 1.5×10^5^ cells/well and 48 hours later incubated in cell culture supernatants collected from pFUGW or pFUW-sTGFβRIIFc-transfected HEK293 cells, in the absence or presence of 5 ng/mL TGFβ for 30 min in a tissue culture incubator at 37°C. Cell lysates were analyzed by western blot, using anti-pSMAD2 (Ser465/467) rabbit mAb (E8F3R, Cell Signaling) followed by anti-rabbit-HRP secondary antibody (Invitrogen), and anti-SMAD2/3 (610842, Becton Dickinson Transduction Laboratories) mouse antibody followed by anti-mouse-HRP secondary antibody (Invitrogen).

### Taqman qPCR and vector copy number analysis

Isolation of RNA from mouse brain tumor tissue, cDNA synthesis, qPCR, isolation of gDNA from mouse bone marrow and vector copy number (VCN) analysis were performed as previously described.^17^ The following custom Taqman primer/probe reagents were used: msTgfbr21_Fwd: (5′-aagagtgcaacgattacatca-3′), msFc1_Rev: (5′- tcagagtaatggtgagcacat-3′), and FAM-labeled Taqman probe (Tgfbr2Fc_1Taqman: 5′-ctgttgatatcggccatggttaga-3′). Gapdh (Mm99999915_g1) was from ThermoFisher Scientific.

### Immunofluorescence and H&E staining

Immunofluorescence on formalin-fixed paraffin-embedded tissue (5 µm sections) using heat-mediated antigen retrieval with EDTA (pH 8.0), immunofluorescence microscopy and image acquisition were performed as described.^17^ Anti-CD45-PE (30-F11; Biolegend) and anti-MMP14 (GeneTex) primary antibodies, followed by anti-rabbit-Alexa488 (Invitrogen) and anti-rat-Cy3 (Jackson ImmunoResearch) secondary antibodies were used on mouse tissue. Anti-human-CD68 antibody (PG-M1; DAKO), and a secondary anti-mouse 488 antibody (Invitrogen) were used on human tissue. H&E staining was performed using Mayer’s hematoxylin solution and eosin Y solution (Sigma).

For quantification of CD68+ cells in human GBM samples, the number of total cells based on nuclear 4',6-diamidino-2-phenylindole (DAPI) stain and the number of CD68+ cells were counted in three fields per sample.

### Statistical analysis

Type of statistical analysis applied is specified in each corresponding figure legend. Statistical analysis was performed using GraphPad Prism V.8 software.

## Results

### Accumulation of myeloid cells in GBM

**Figure 1:**
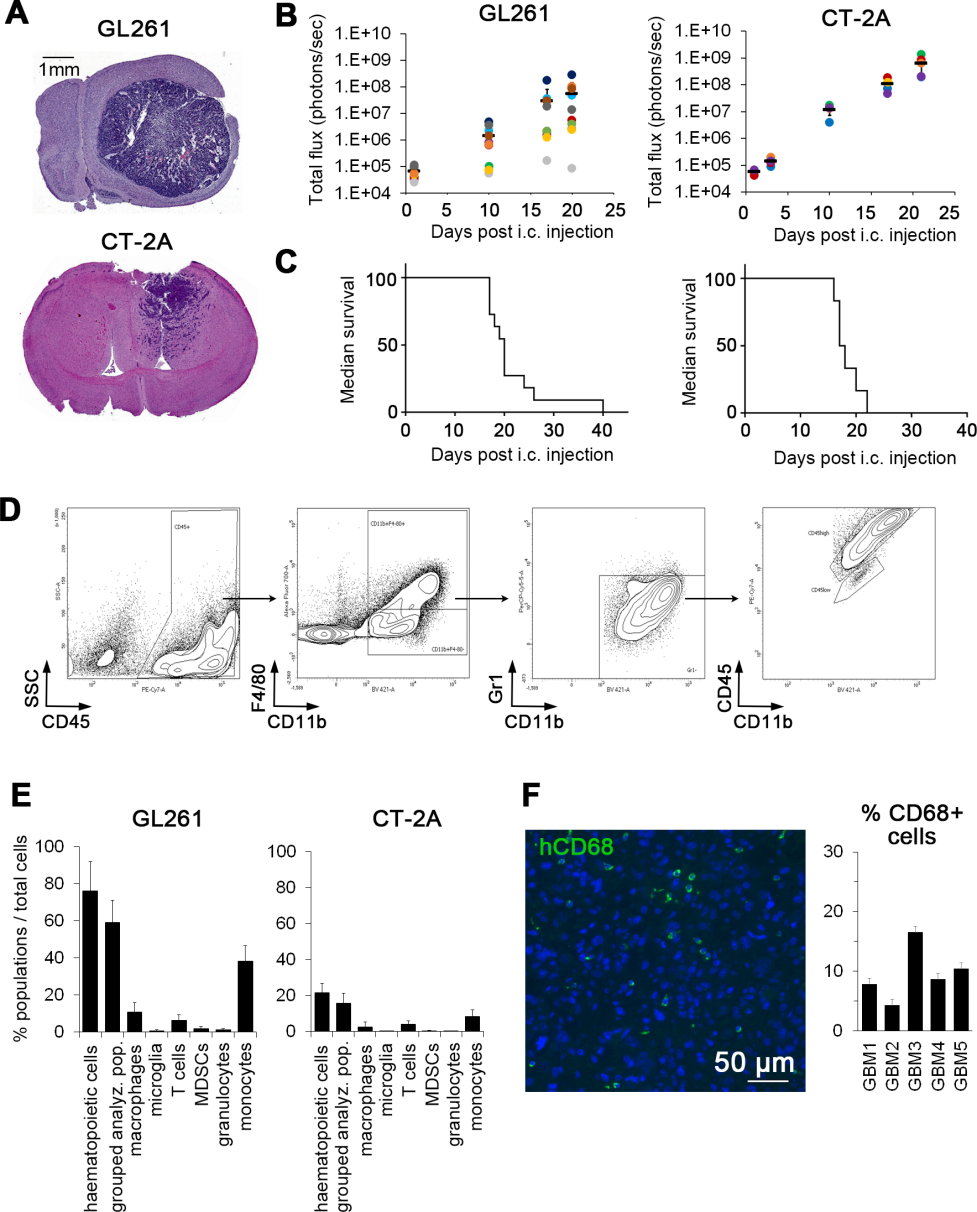
Accumulation of myeloid cells in intracranial glioblastoma (GBM). (A) H&E staining of coronal mouse brain sections showing tumors generated from GL261 and CT-2A cancer cells. (B) GL261/Fluc and CT-2A/Fluc cells (1×10^5^), respectively, were implanted intracranially (i.c.) and tumor growth was monitored by bioluminescence imaging (n=10 for GL261; n=5 for CT2A). (C) Survival curves for GL261 (n=11) and CT2A (n=6) cancer models. (D) Immune cell infiltration in GL261 and CT-2A brain tumors was determined by flow cytometry in mice with engrafted GFP+ hematopoietic stem cells (HSCs) from C57Bl/6-Tg(UBCGFP)30Scha/J transgenic mice. Representative contour plots and the gating strategy employed to identify macrophages (CD11b^+^F4/80^+^Gr1^-^CD45^high^) and microglia (CD11b^+^F4/80^+^Gr1^-^CD45^low^) are shown. (E) Quantification of tumor-infiltrating immune cells in GL261 (n=5) and CT-2A tumors (n=5) by flow cytometry. Hematopoietic cells (CD45^+^), grouped analyzed cell populations (granulocytes, monocytes, macrophages, myeloid-derived suppressor cells (MDSCs), microglia, T-cells), macrophages (CD11b^+^ F4/80^+^CD45^high^), microglia (CD11b^+^F4/80^+^Gr1^-^CD45^low^), T cells (CD45^+^CD11b^-^CD3^+^), MDSCs (CD11b^+^F4/80^-^Gr1^+^), granulocytes (CD11b^+^F4/80^-^Ly6G^+^) and monocytes (CD11b^+^F4/80^-^Ly6C^+^) were quantified in mice with engrafted GFP+ HSCs from C57Bl/6-Tg(UBCGFP)30Scha/J transgenic mice. Error bars represent SD. (F) Infiltration of CD68+ macrophages/microglia in patient GBM tissue as detected by immunofluorescence. Nuclei are stained with DAPI. Scale bar, 50 µm. Graph to the right shows the quantification of CD68+ cells in patient GBM tumors (n=5). DAPI, 4',6-diamidino-2-phenylindole.

Following their intracranial implantation in C57Bl6 mice, firefly luciferase (Fluc)-tagged GL261/Fluc and CT-2A/Fluc GBM cells formed large lesions within approximately 3 weeks (figure 1A–C). A pronounced infiltration of cancer cells into the surrounding tissue in CT-2A model mimicked the infiltrative nature of human GBM (figure 1A). Analysis of tumor-infiltrating immune cells by flow cytometry (figure 1D) identified marked infiltration of CD45+ hematopoietic cells, amounting to 75.96%±15.72% of all cells in GL261 model, and 21.68%±5.33% in the CT-2A model. The predominant cell population were CD11b+ myeloid cells (45.95%±7.24% and 7.81%±4.10% of all cells in GL261 and CT-2A models, respectively), containing mainly blood-derived macrophages and monocytes, and only low levels of microglia, granulocytes, and myeloid-derived suppressor cells (below 2% of all cells) (figure 1E). T cell infiltration ˂4% was observed in both models (figure 1E). Despite differences in myeloid cell infiltration between the GL261 and CT2A models, no significant difference in survival was detected (median survival was 20±6.74 days for GL261, and 17.5±2.25 days for CT2A model; figure 1C). In line with previous reports,^21^ tumor-infiltrating CD68^+^ microglia/macrophages could also be detected in human patient samples (n=5; 4.3% to 16.5% of all cells) (figure 1F). Due to the pronounced infiltration of myeloid cells into both mouse and human GBM, we reasoned that myeloid cells derived from HSCs may be used in cell therapy approaches.

### Homing of genetically modified HSC progeny to GBM

**Figure 2:**
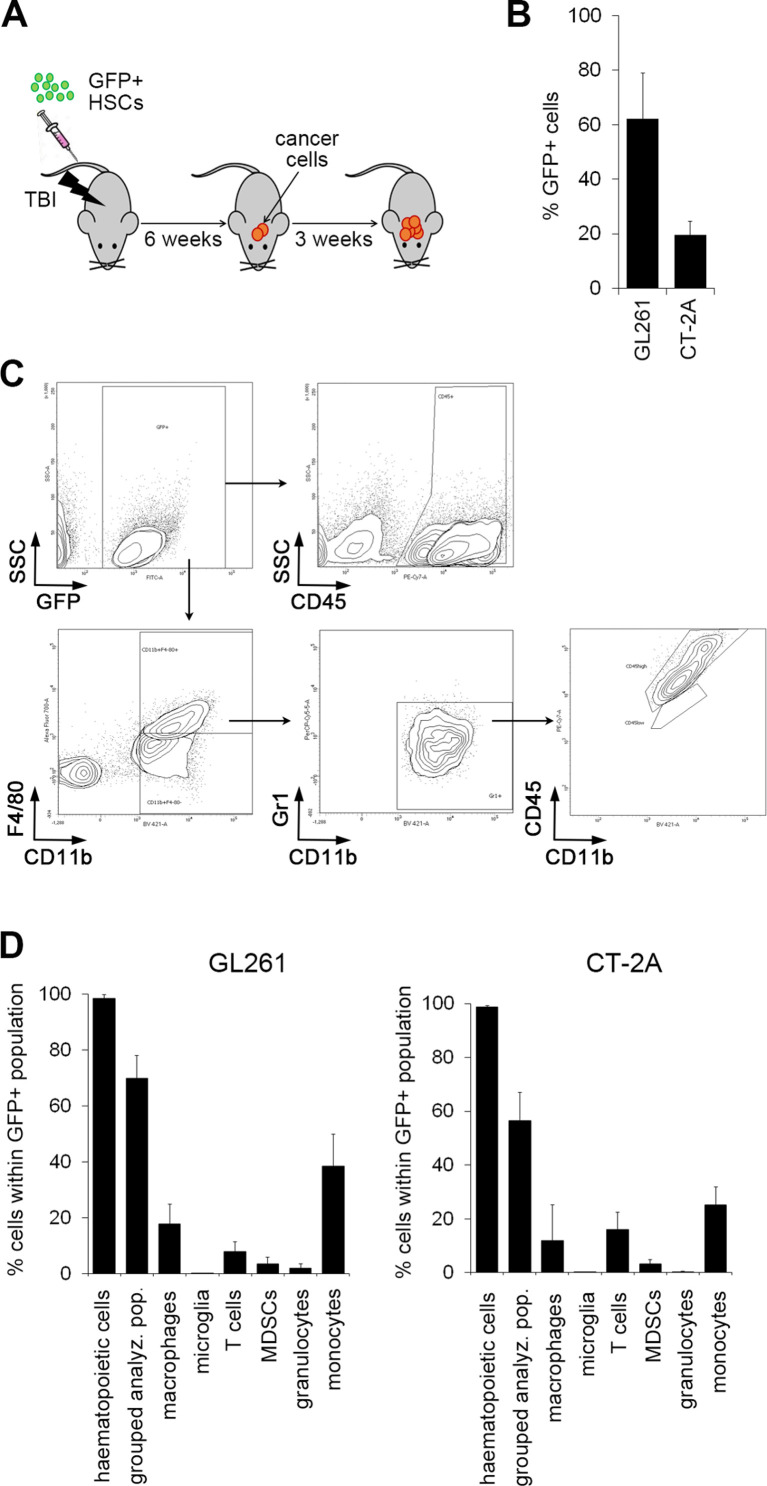
Homing of genetically modified hematopoietic stem cell (HSC) progeny to glioblastoma. (A) Experimental scheme: green fluorescent protein (GFP)-tagged HSCs isolated from C57Bl/6-Tg(UBCGFP)30Scha/J transgenic mice were injected intravenously into lethally irradiated C57Bl/6J mice (TBI; total body irradiation). Following bone marrow reconstitution, GL261 or CT-2A cancer cells were injected intracranially and immune cell infiltration was assessed by flow cytometry at ~3 weeks post-cancer cell injection. (B) The percentage of GFP^+^ HSC progeny infiltrating brain tumors generated from GL261 (n=5) or CT-2A (n=5) cancer cells was determined by flow cytometry. (C) Representative contour plots and the gating strategy employed to identify macrophages (CD11b^+^F4/80^+^Gr1^-^CD45^high^) and microglia (CD11b^+^F4/80^+^Gr1^-^CD45^low^) within the GFP^+^ cell population are shown. (D) Quantification of cell populations within GFP^+^ HSC progeny infiltrating GL261 (n=5) and CT-2A tumors (n=5) by flow cytometry. Hematopoietic cells (CD45^+^), grouped analyzed cell populations (granulocytes, monocytes, macrophages, myeloid-derived suppressor cells (MDSCs), microglia, T cells), macrophages (F4/80^+^CD11b^+^CD45^high^), microglia (CD11b^+^F4/80^+^Gr1^-^CD45^low^), T cells (CD45^+^CD11b^-^CD3^+^), MDSCs (CD11b^+^F4/80^-^Gr1^+^), granulocytes (CD11b^+^F4/80^-^Ly6G^+^) and monocytes (CD11b^+^F4/80^-^Ly6C^+^) were quantified. Error bars represent SD.

To provide a proof-of-principle for lentiviral HSC gene therapy targeting GBM, GFP+ murine HSCs isolated from transgenic C57Bl/6-Tg(UBCGFP)30Scha/J mice were transplanted into lethally irradiated C57Bl6 mice. Intracranial tumors were analyzed 3 weeks following the intracranial implantation of GL261 and CT-2A cancer cells, respectively (figure 2A). GFP^+^ cells represented 62.11%±16.73% (GL261 model) and 19.52%±5.15% (CT-2A model) of all cells within tumors (figure 2B). The majority of GFP^+^ cells were CD45^+^CD11b^+^ myeloid cells (57.33%±6.53% and 37.43%±16.28% in GL261 and CT-2A models, respectively), consisting mainly of macrophages/monocytes, with very low representation of granulocytes and myeloid derived suppressor cells (below 3.2%). As expected, microglia were not among GFP^+^ cells, as they are derived from the yolk sac rather than HSCs^17 22^ (figure 2C, D).

### Delivery of TGFβ-targeting blocking peptide to GBM in HSCs under a myeloid cell-specific gene promoter

**Figure 3:**
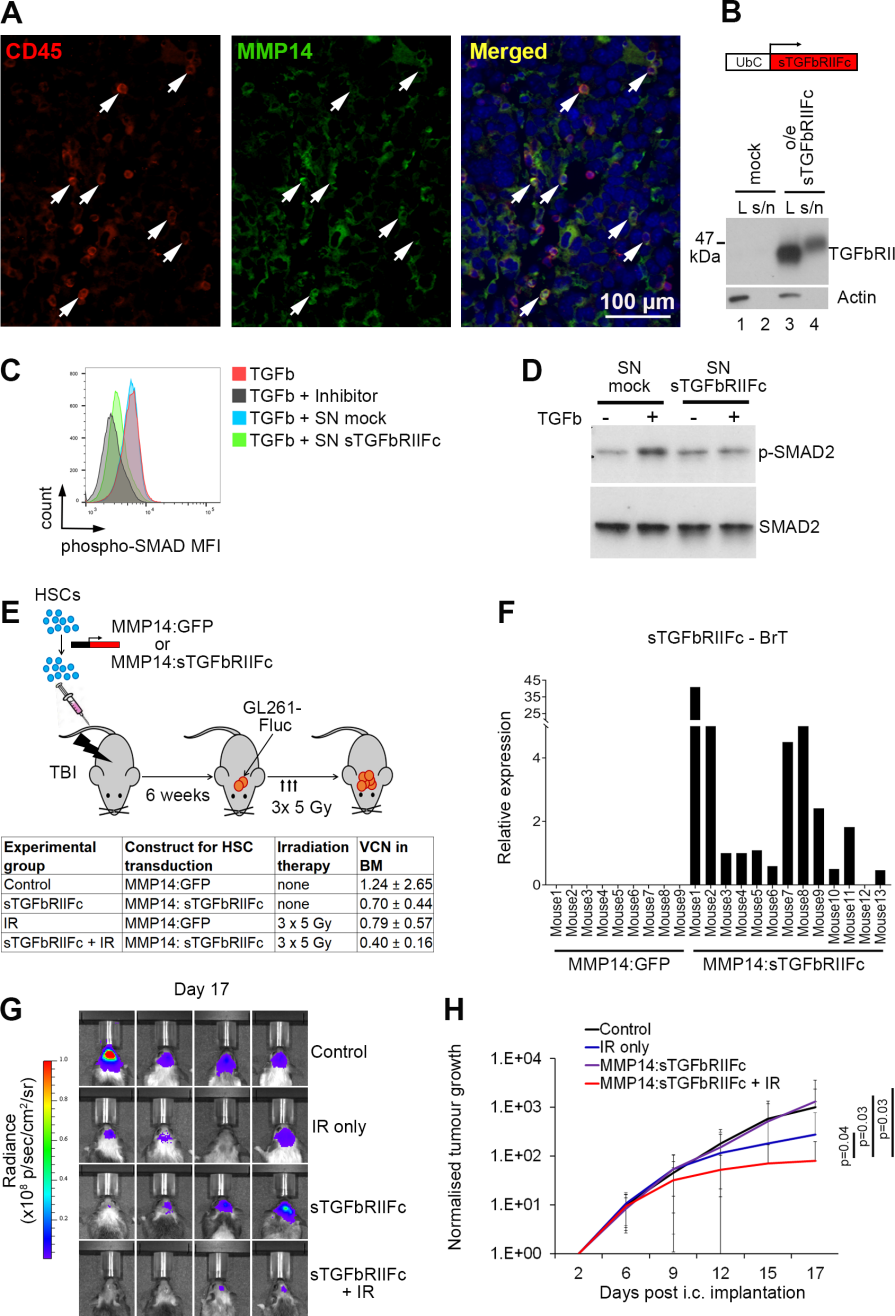
Delivery of transforming growth factor beta (TGFβ)-targeting therapy to glioblastoma in hematopoietic stem cells (HSCs) under a myeloid cell-specific gene promoter, with and without irradiation (IR) therapy. (A) Expression of matrix metalloproteinase 14 (MMP14) in tumor-infiltrating CD45^+^ hematopoietic cells (white arrows) in intracranial GL261 glioblastoma model as determined by immunofluorescence. Levels for the green channel were adjusted using Adobe Photoshop, applying equal adjustment to all images. Scale bar: 100 µm. (B) Western blot analysis of sTGFβRIIFc expression in the cell lysate (L) and cell culture supernatant (s/n) of HEK293 cells transiently transfected with a lentiviral vector expressing sTGFβRIIFc under the Ubiquitin C (UbC) promoter. Untransfected HEK293 cells were used as a control (mock). (C) Quantification of phospho-SMAD2/3 mean fluorescence intensity (MFI) in OT-I T cells following stimulation with TGFβ (5 ng/mL) for 30 min, alone or in the presence of TGFβ inhibitor SB431542, cell culture supernatant collected from pFUGW-transduced HEK293 cells (SN mock) or supernatant collected from the pFUW-sTGFbRIIFc-transduced HEK293 cells (SN sTGFbRIIFc). One representative repeat out of 3 is shown. (D) Western blot showing phospho-SMAD2 and total SMAD in HeLa cells following 30 min incubation in cell culture supernatants collected from pFUWG or pFUW-sTGFbRIIFc-transduced HEK293 cells (SN mock and SN sTGFbRIIFc, respectively), in the presence or absence of TGFβ (5 ng/mL) as indicated. One representative repeat out of 3 is shown. (E) Top: experimental scheme: HSCs transduced with a lentiviral construct expressing sTGFβRIIFc under MMP14 promoter fragment (MMP14:sTGFβRIIFc) or a control construct expressing GFP under MMP14 promoter fragment (MMP14:GFP) were transplanted into lethally irradiated C57Bl6 mice (TBI; total body irradiation). Following bone marrow reconstitution 6 weeks later, GL261 glioblastoma cells were implanted intracranially. Three doses of IR therapy (5 Gy each) were administered on days 7, 8 and 9 post-tumor implantation in 2 of the experimental groups. Bottom: a table summarizing experimental groups, with indication of average vector copy number (VCN) in the bone marrow (BM; n=4/4/4/7 for control, IR, sTGFβRIIFc, and sTGFβRIIFc+IR combination therapy, respectively). (F) Quantification of sTGFβRIIFc expression in brain tumors (BrT) by qPCR. Relative expression is shown. (G) Representative bioluminescence images of mice from the four experimental groups as indicated (day 17 post-intracranial implantation of cancer cells). (H) Quantification of tumor growth via bioluminescence imaging. The signals for each day were normalized to the signals at day 2 post-cancer cell implantation. Error bars represent SD. Statistical significance was determined by one-tailed t-test on day 17 (p values as indicated). n=11/11/10/16 for control, IR, sTGFβRIIFc, and sTGFβRIIFc+IR combination therapy, respectively (combined data from two independent experiments).

We have recently demonstrated that MMP14 is highly expressed in the myeloid progeny of HSCs infiltrating brain metastases. An ~2 kb MMP14 promoter fragment demonstrated specific activity in brain metastases-infiltrating myeloid cells as compared with the myeloid cells isolated from other tissues, and resulted in a specific delivery of therapeutic molecules to brain metastases.^17^ We here confirmed that MMP14 is also expressed in hematopoietic cells infiltrating syngeneic GBM (figure 3A), providing a rationale for using MMP14 promoter fragment to drive the expression of therapeutic genes in GBM models.

TGFβ is a major driver of glioma progression.^5 6^ To inhibit TGFβ, we decided to use HSC gene therapy to deliver soluble TGFβ decoy receptor fused to the fragment crystallisable IgG (Fc) region (sTGFβRIIFc)^20^ to intracranial GL261 tumors. The expression and secretion of sTGFβRIIFc was first confirmed under the Ubiquitin C promoter in vitro in HEK293 cells (figure 3B). The functionality of the construct was confirmed by demonstrating an inhibition of TGFβ-induced SMAD2/3 phosphorylation in cell culture supernatants collected from pFUW-sTGFβRIIFc-transduced as compared with the pFUGW-transduced HEK293 cells. This was demonstrated in OT-I T cells (figure 3C) as well as in HeLa cells (figure 3D). sTGFβRIIFc was then cloned downstream of our previously characterized MMP14 promoter fragment.^17^ This vector was used for lentiviral transduction of murine HSCs prior to their transplantation into recipient mice (figure 3E). Notably, irradiation (IR) is known to increase TGFβ expression in glioma.^7 10^ This provided a rationale to combine the TGFβ blocking therapy with localized IR to the tumor, and test it in parallel to the respective monotherapies (sTGFβRIIFc and IR) and a control group. Mice in IR and control groups received HSCs transduced with a lentiviral construct expressing GFP downstream of MMP14 promoter (figure 3E).

A successful delivery of sTGFβRIIFc to intracranial GBM was confirmed by qPCR (figure 3F). Tumor growth was significantly decreased in the combination therapy group as compared with the control group (p=0.03), sTGFβRIIFc monotherapy (0=0.03) and IR (p=0.04) (figure 3G, H). This demonstrated that sTGFβRIIFc can be delivered to GBM using HSC gene therapy, and this significantly improved the efficacy of IR therapy.

### Combined HSC gene therapy targeting TGFβ and IR result in long-term protection against intracranial GBM

**Figure 4:**
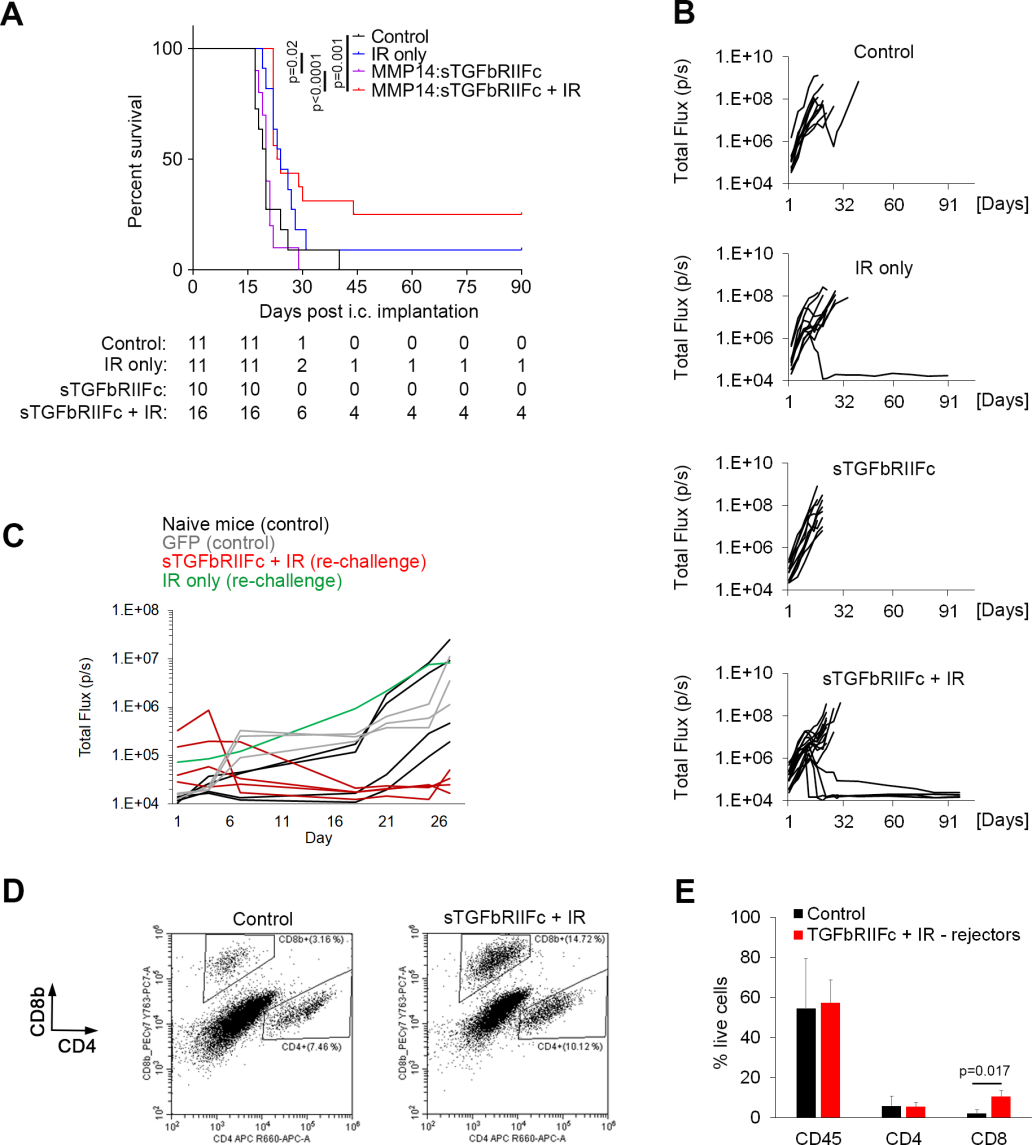
Hematopoietic stem cell (HSC) gene therapy targeting transforming growth factor beta (TGFβ) in combination with irradiation (IR) results in a long-term protection against intracranial glioblastoma. (A) Survival of mice bearing GL261 intracranial tumors. The table below the graph shows group sizes at the beginning (day 0) and number of animals at risk (eg, animals that are alive) for indicated days. Pooled data from two independent experiments are shown. Statistical significance was determined by two-tailed log-rank test. (B) Growth curves for individual tumors as obtained by bioluminescence imaging, displaying total flux in photons per second (p/s). (C) Mice that rejected GL261 tumors following the first intracranial implantation of cancer cells plus therapy were rechallenged by a second intracranial implantation of GL261 cells at 90 days post-tumor rejection (red and green lines). Mice that have received transplantation of MMP14:GFP-transduced HSCs (gray lines) or naïve mice (black lines) were implanted with GL261 cells intracranially for the first time and used as controls. Tumor growth was quantified by bioluminescence imaging as in (B). (D and E) Representative dot plots showing analysis of CD8+ and CD4+ T cells (D) and quantification of immune cells (E) in intracranial tumors (control mice as specified in C) or brain area corresponding to the cancer cell implantation site (mice that have rejected tumors for the second time in the combination therapy group) by flow cytometry at 3 weeks post-cancer cell implantation. Statistical significance was determined by two-tailed t-test (n=3 and 4, respectively, in first and second experiment for control group; n=2 per experiment for rechallenged long-term survivors from the combination therapy group).

Survival of mice was monitored to assess long-term benefits of the therapy. Only the combination therapy resulted in a significantly longer survival time as compared with the control group (p=0.0016; two-tailed log-rank test) and TGFβ-blocking monotherapy (p<0.0001; two-tailed log-rank test) (figure 4A). Moreover, 25% of the mice (4 out of 16) in the combination therapy group demonstrated long-term survival with a complete tumor rejection, as compared with only 1 out of 11 mice (9 %) in the IR group and 0% in the remaining groups (figure 4A, B). At 90 days post-tumor rejection, the surviving mice were rechallenged with tumors through intracranial implantation of GL261 cells. While the tumors grew efficiently in naïve control mice and in the rechallenged mouse from the IR group, the tumors failed to grow in all four rechallenged mice from the combination therapy group (figure 4C). This demonstrated that in contrast to the IR monotherapy, the combination therapy resulted in a long-term protection against cancer.

Analysis of immune cells at the tumor implantation site at 3 weeks post-intracranial cancer cell injection revealed a significantly higher proportion of CD8+ T cells in the four mice from the combination therapy group that have rejected tumors for the second time, as compared with the control mice with successfully growing tumors. In contrast, the infiltration of total CD45+ hematopoietic cells and CD4+ T cells was similar between the two groups (figure 4D, E). This further suggests that, in addition to significantly reducing tumor burden and prolonging survival, TGFβ-blocking HSC gene therapy in combination with IR provided a long-term protection against cancer through the development of memory T cell responses in ¼ of the mice.

## Discussion

Lentiviral gene transfer has recently demonstrated an excellent safety record, with promising results in patients.^15 16^ The advantage of HSCs over other commonly explored stem cell types, such as mesenchymal and neuronal stem cells,^12^ is the ability to isolate HSCs in large quantities, and well-established procedures for their therapeutic use. HSC-derived myeloid cells, mostly consisting of macrophages, can account for over 50% of all cells in GBM,^21 23^ making them highly suitable therapeutic delivery vehicles in this context. As myeloid cells are ubiquitously present in the body, we here used a vector with MMP14 promoter^17^ to deliver TGFβ-blocking therapy specifically to experimental brain tumors, and thereby provided a proof-of-principle for the efficacy of HSC gene therapy targeting GBM using a tumor myeloid cell-specific gene promoter.

A synergy between IR and TGFβ blockade, using therapeutic approaches other than gene therapy, has been previously reported.^7–10^ In line with this, in our study TGFβ-blocking HSC gene therapy combined with IR significantly reduced tumor burden as compared with monotherapies. Notably, among other suppressive effects on the immune system, TGFβ has been shown to suppress the cytotoxic function of CD8+ T cells in cancer,^24^ and TGFβ inhibition has been shown to enhance tumor elimination by T cells.^25^ Thus, the efficacy of combined TGFβ blockade and IR in our model is likely a combination of direct effects on cancer cells and boosting of antitumor immunity.

Durable memory responses against cancer observed in ¼ of mice in the combination therapy group in our model correlated with a significant increase in CD8+ T cells at the tumor implantation site in tumor rechallenged mice as compared with naïve mice challenged with tumors for the first time. In line with this, gene expression signatures associated with IFNγ and immune-mediated rejection were previously observed in breast cancer models treated with IR and antibody-mediated TGFβ blockade, but not in monotherapy-treated tumors.^9^ A subsequent clinical study comparing two doses of fresolimumab in combination with focal radiotherapy to a metastatic site reported a strong increase in the CD8+ central memory T cells with a higher fresolimumab concentration.^8^ Thus, it is likely that TGFβ blockade in combination with IR enhances both effector T cell function during initial tumor challenge, as well as long-term memory response.

In the context of TGFβ-blocking therapy in GBM, the advantage of our approach is that (1) it overcomes the problem of penetration through the blood–brain barrier, allowing for an efficient delivery of TGFβ blockade with a potential to also reach tissue-invading GBM cells; (2) as transgene expression under MMP14 promoter is restricted to intratumoral myeloid cells,^17^ systemic side effects such as keratoacanthomas^8^ are expected to be minimized. Use of tumor-myeloid cell-specific rather than general myeloid promoter is also important to prevent systemic effects on the immune cells; for example, exacerbated colitis with proinﬂammatory cytokine production has been reported in a mouse colitis model expressing dominant negative TGFβRII downstream of a general macrophage promoter.^26^ Despite the use of our tumor myeloid cell-specific promoter, potential off-target toxicities cannot be excluded and would need to be closely monitored in the context of clinical translation.

The extent of myeloid cell infiltration into glioma is variable in patients and this is also reflected in our models, with higher percentage of myeloid cells in GL261 (~40%) as compared with CT2A tumors (~8%). Notably, extent of myeloid cell infiltration in individual patients is also expected to have an impact on the therapeutic efficacy, and strategies to enhance myeloid cell homing to tumors may be considered.

In our study, we used a strong myeloablative irradiation conditioning to deplete HSCs in the bone marrow and make space for the injected genetically modified HSCs. For clinical translation, it will be critical to optimize HSC transduction protocols and the conditioning regimen, in order to balance a need to achieve therapeutic levels of transgene while minimizing toxicity. Busulfan is most commonly used for conditioning in the context of autologous HSC gene therapy in genetic disorders, and different busulfan doses have been used to achieve optimal intensity of conditioning and appropriate levels of HSC engraftment tailored to each respective disease.^27^ HSCs have not yet been used in the clinic to deliver therapies targeting brain tumors directly. However, HSC gene therapy to overexpress mutant methylguanine methyltransferase (MGMT) has been used for bone marrow chemoprotection during temozolomide treatment in combination with O^6^-benzylguanine, the inhibitor of MGMT, to permit dose escalation, resulting in surpassing of the median survival for patients with GBM with poor prognosis.^28^ In this study, conditioning with carmustine resulted in successful HSC engraftment. This demonstrates the feasibility of applying HSC gene therapy in patients with brain tumor and indicates an opportunity for clinical translation of our therapeutic approach.
